# Stent-Graft Removal and Extra-Anatomical Bypass for the Treatment of Stent-Graft Infection after Endovascular Aneurysm Repair

**DOI:** 10.3400/avd.cr.21-00106

**Published:** 2022-03-25

**Authors:** Naoya Kuriyama, Atsuhiro Koya, Shinsuke Kikuchi, Daiki Uchida, Nobuyoshi Azuma

**Affiliations:** 1 Department of Vascular Surgery, Asahikawa Medical University, Asahikawa, Hokkaido, Japan

**Keywords:** infected aneurysm, stent-graft infection, extra-anatomical bypass

## Abstract

Stent-graft infection is a rare but potentially life-threatening complication of endovascular aortic repair. There are currently no consensus guidelines for treating stent-graft infections, but surgical treatment is generally considered preferable due to the low overall survival rate of patients receiving conservative therapy; however, the revascularization method remains controversial. We report a case in which stent-graft infection after endovascular aneurysm repair was successfully treated by stent-graft removal and extra-anatomical bypass (EAB). EAB is an effective method of revascularization for stent-graft infection.

## Introduction

Endovascular aneurysm repair (EVAR) is an effective treatment method for abdominal aortic aneurysm (AAA) rupture and impending rupture, infected AAA, etc.^1)^ Among post-EVAR AAA-related complications, endoleak and graft infection are the most important. The frequency of post-EVAR stent-graft infection is low at 0.2%–0.7%.^2)^ However, the complications are life-threatening, with the mortality rate within 30 days after surgery being approximately 17%.^3)^ The treatment strategies for stent-graft infection are selected from among conservative methods such as antibacterial agent administration, drainage, lavage, and surgical methods involving extra-anatomical bypass (EAB) or in situ bypass.^4)^

We report the case of a patient whose stent-graft infection after EVAR for impending AAA rupture was treated by EAB.

## Case Report

**Figure 1:**
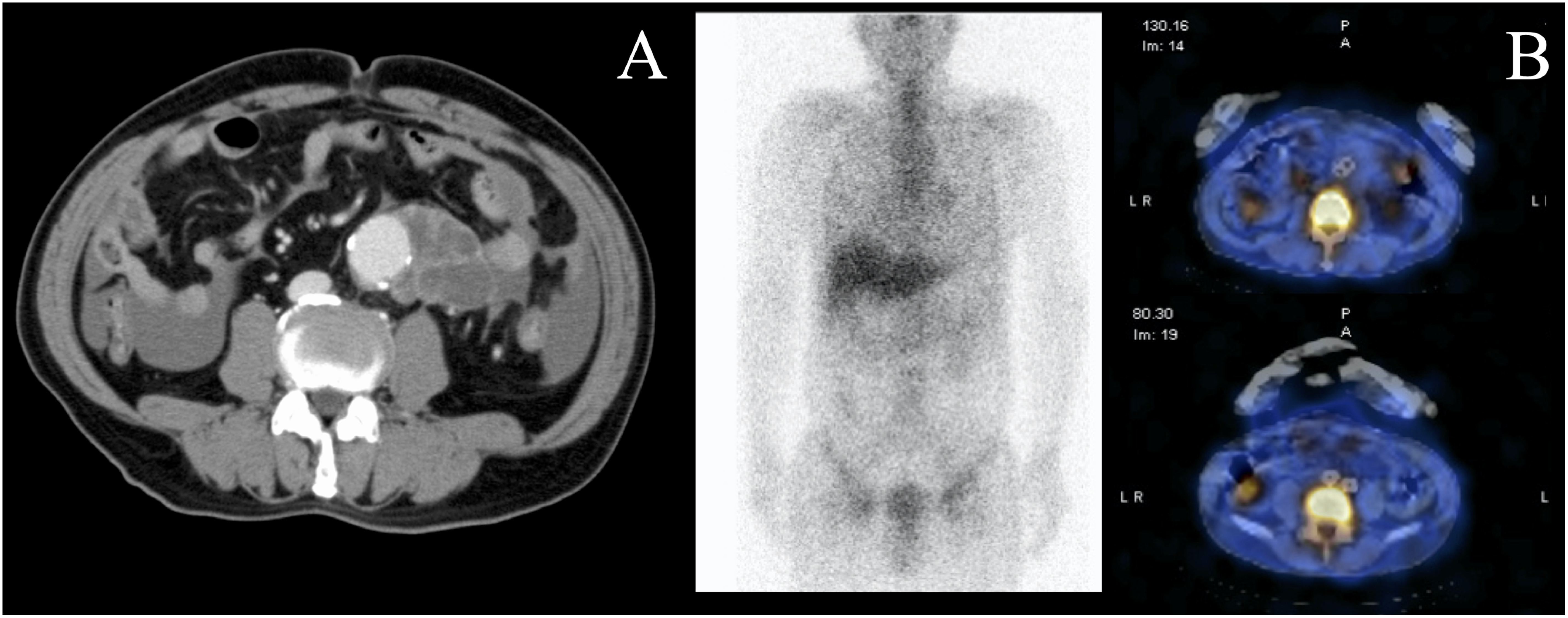
Fig. 1 An enhanced computed tomography (CT) and Ga-67-citrate scintigraphy before endovascular aortic repair. (**A**) CT showed an abdominal aortic aneurysm (AAA) surrounded by diverse cystic structures up to 52 mm in diameter. (**B**) A Ga-67-citrate scintigraphy showed no significant uptake of Ga-67-citrate around the AAA and stent-graft.

**Figure 2:**
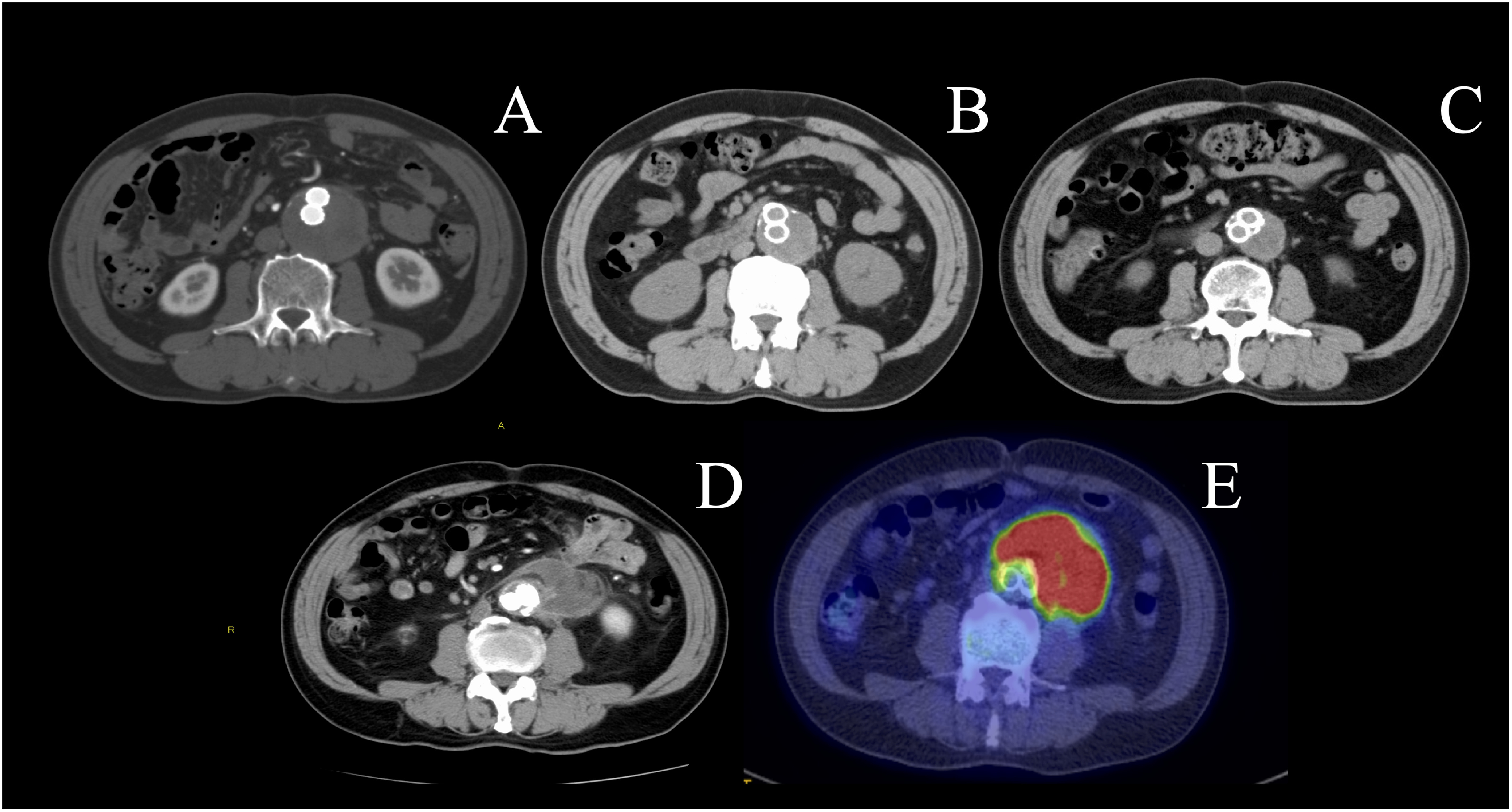
Fig. 2 Computed tomography (CT) showed the postoperative change of the aneurysm. (**A**) A half year after endovascular aortic repair, the diameter of the aneurysm was 47 mm. (**B**) The diameter of the aneurysm was 34 mm 2 years after endovascular aortic repair (EVAR). (**C**) The diameter of the aneurysm was 32 mm 3 years after EVAR. (**D**) At 3.5 years after EVAR, an enhanced CT showed a lobulated lesion around the stent-graft. (**E**) A positron emission tomography-CT showed a high ^18^F-fluorodeoxyglucose uptake of the aneurysm.

The patient was a 64-year-old man. At the age of 61 years, magnetic resonance imaging was performed as part of a detailed examination by a local physician for low back pain. Impending AAA rupture was diagnosed, and the patient was urgently transferred to the authors’ hospital. Enhanced computed tomography (CT) was performed, and it revealed an AAA surrounded by diverse cystic structures up to 52 mm in diameter (**Fig. 1A**). Emergency EVAR was performed using a Gore Excluder (W. L. Gore and Associates, Flagstaff, AZ, USA), followed by administration of ampicillin/sulbactam (1.5 g) for 11 days at 12-hour intervals. Infectious AAA was suspected by preoperative CT. Preoperative blood culture was not harvested because of emergency surgery. However, blood cultures immediately after EVAR were negative. In addition, Ga-67-citrate scintigraphy was performed (**Fig. 1B**). However, no significant uptake was found; after 11 days, the patient was discharged. Administration of levofloxacin (500 mg/day) was then continued on an outpatient basis. The aneurysm diameter decreased progressively, and the cystic structures disappeared (**Figs. 2A**–**2C**).

At 3.5 years after EVAR, the patient repeatedly experienced low back pain and fever at over 39°C. He was hospitalized at our hospital. Hematology tests showed elevated leukocyte count and C-reactive protein (CRP) concentrations at 13400 cells/µL and 21.5 mg/dL, respectively. Contrast CT showed an enlarged mass opacity below the renal artery surrounding the stent-graft (**Fig. 2D**). Furthermore, it showed no obvious bone destruction and no fluid retention in the intervertebral discs. After admission, administration of meropenem (1 g) at 8-hour intervals was initiated; however, the elevated leukocyte count, CRP, and back pain persisted. At admission, the blood culture results were negative. The performance of ^18^F-fluorodeoxyglucose positron emission tomography (FDG-PET) showed a marked uptake of FDG, consistent with the mass lesions shown by CT (**Fig. 2E**). Stent-graft infection was suspected; therefore, we decided to perform surgery.

**Figure 3:**
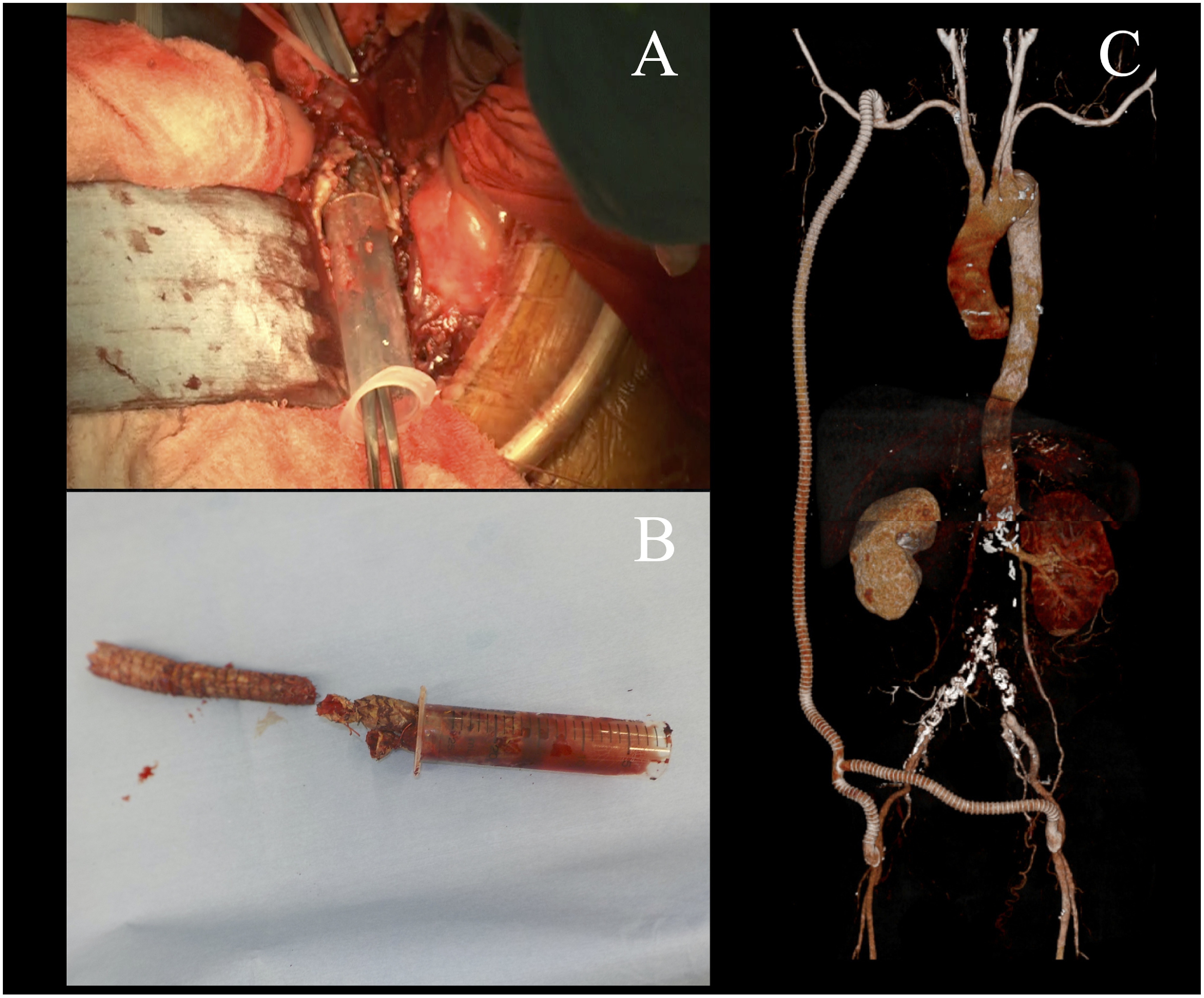
Fig. 3 (**A**) The infected stent-graft was removed using a 20-milliliter syringe. (**B**) Removed infected stent-graft. (**C**) A postoperative three-dimensional computed tomography showed the right axillary-bifemoral bypass.

Under general anesthesia, using an expanded polytetrafluoroethylene graft with a T-shaped, 8-millimeter ring attached (Gore-Tex Vascular Graft; W. L. Gore and Associates), a right axillary-bifemoral bypass was created to ensure blood flow to the legs and intrapelvic organs. We closed and draped the skin incision of the bypass, followed by laparotomy. After taping using a 4-millimeter vessel loop and clamping, the abdominal aorta was severed, and the stent-graft was exposed. Following ligation and dissection of both legs of the stent-graft, the legs were pulled out of the left and right common iliac arteries, and each of these arteries was ligated. Next, the tip of the 20-milliliter syringe was cut off, and the main body of the stent-graft was inserted inside the syringe (**Fig. 3A**). The syringe was pushed inside while gradually releasing the clamp on the abdominal aorta. To minimize hemorrhage associated with removal, while the main body was being pulled free, the abdominal aorta was kept closed with a vessel loop and tourniquet, and the abdominal aorta was clamped again. The abdominal aorta was then closed as an aortic stump using horizontal mattress sutures with tachosil (Takeda Austria GmbH, Linz, Austria) as a pledget with 4-0 prolene. The lumbar arteries were handled by suture closure from the inside of the aorta. Finally, the aortic wall has been left, but the surrounding tissue has been carefully debrided and cleaned as much as possible, followed by abdominal closure. The pathological sample was not collected; however, a partial abscess was formed in the aortic wall, and the fluid was examined by Gram staining during surgery. It revealed that no bacteria were detected, but many neutrophils were observed.

After surgery, 1 g of meropenem was administered at 8-hour intervals for 16 days. After surgery results, the culture for blood, the wall of the aorta, the iliac artery, and the extracted stent-graft (**Fig. 3B**) were negative. The postoperative three-dimensional CT is shown in **Fig. 3C**. The immunoglobulin G4 (IgG4) level was 24.2 mg/dL; thus, IgG4-related vasculitis was eliminated. By 16 days after surgery, the leukocyte count and CRP level had approximately normalized, and the patient was discharged in an ambulatory state. After discharge, oral levofloxacin (500 mg/day) was initiated, and the patient was monitored for 3 years, although no recurrence or infection was found.

## Discussion

Although ruptured AAA is lethal without early intervention, EVAR or open surgical repair (OSR) treatment is controversial. A recent systematic review found no difference in mortality between the two groups, but the EVAR group was reported to have less bowel ischemia.^1)^ Infected AAA is rare but fatal and should also require surgical intervention. Recently, EVAR has been attracting attention as a treatment for infected AAA. Hosaka et al. showed that there is no significant difference in the mortality rate between OSR and EVAR.^5)^ Thus, it has been suggested that EVAR may be effective for both ruptured AAA and infected AAA. However, they also showed that the eradication of infection following EVAR was limited compared with OSR. Thus, we selected EVAR for life saving and prevention of rupture of AAA since AAA was symptomatic and suspected impending rupture. On the other hand, as shown in the current case, surgical treatment for recurrent aneurysm-related infection was clinically important.

Stent-graft infection is diagnosed comprehensively based on clinical symptoms, contrast CT, and culture with samples collected from the blood, the stent-graft, and the drainage from around the aorta.^6)^ *Staphylococcus* was the most common causative bacteria at 30.1%, followed by *Streptococcus* at 14.8%. In approximately 27%–30% of patients, the culture results are negative due to preoperative administration of antibacterial agents.^3,6)^ Recently, FDG-PET has gained attention as a useful diagnostic method for stent-graft infection; nevertheless, no standards for this have been established to date. No clear diagnostic criteria for stent-graft infection have been established,^6)^ and it is not unusual for diagnosis to be challenging. This patient was administered antibacterial agents for 7 days before surgery, and the blood and stent-graft culture results were negative. However, there was no improvement in the initial physical examination or inflammatory response.

Additionally, FDG-PET at the same sites showed significant FDG uptake; therefore, we diagnosed graft infection. Since the patient was 64 years old and relatively young, we believed that conservative treatment alone might cause repeated infections in the future; therefore, we opted for surgical intervention. Recently, gene amplification and sequencing have been established as a method for detecting and identifying microorganisms. Such methods may also be useful in identifying causative organisms. Furthermore, CT-guided puncture culture before the second surgery may have been useful in diagnosing the causative organism.

In this patient, a late stent-graft infection occurred 3.5 years after EVAR. Late infections are typically mild and result from colonization by low-virulence organisms, such as *Staphylococcus epidermidis*.^7)^ In addition, the nature of late infection often lacks the signs of sepsis. The treatment may include long-term administration of antibacterial agents. There is no consensus on the appropriate duration of postoperative antibiotic therapy. A previous report showed that it should be continued for 6 weeks or longer. However, the reinfection occurred 3.5 years after surgery. Laohapensang et al. showed that antibiotic therapy should be continued as life-long therapy.^8)^ Therefore, we believe that oral antibiotics should be continued as long-life therapy in this patient.

There are no consensus guidelines for the treatment of stent-graft infections. According to Li et al., the overall survival rate of patients receiving conservative treatment based on antibacterial agents is only 33%.^3)^ Contrarily, the survival rate of patients who undergo surgical resection is approximately 58%.^3)^ Therefore, surgical treatment is considered preferable if the stent-graft infection is operable.

The surgical techniques used to treat stent-graft infections are broadly divided into stent-graft removal and vascular reconstruction. Stent-graft removal involves risks of vascular wall damage by the stent-graft, and particularly, there are several problems with removing stent grafts that are barbed on the proximal site. A previous report suggested that a method for handling this involves severing the tip region of a 20-milliliter syringe and pulling the stent-graft out simultaneously while inserting it inside the syringe.^9)^ In the present case, although the proximal side of the stent-graft was not barbed, a method to maximally reduce vascular wall damage while pulling the stent-graft out was used, and it was removed safely. Additionally, depending on the condition of the aneurysm, it is a viable option to place the stent-graft lower to allow the space for the aortic clamp when placing a stent-graft for an infected aneurysm. Non-barbed stent-graft, such as Excluder (W. L. Gore and Associates), might be a more appropriate option in anticipation of subsequent stent-graft removal.

Vascular reconstruction such as in situ or EAB is required. Aortic stump formation can be more readily avoided in situ than EAB, and the former also has a higher patency rate.^8)^ However, it necessitates procedures such as omental implantation and is thus not appropriate for severe infection.^7)^ On the other hand, EAB involves concerns about long-term graft patency and risks of aortic stump rupture and renal arterial occlusive complications, but the surgery duration is short^10)^; this method is useful for patients with severe infection around the graft.^8)^ To avoid aortic stump rupture, we should strictly control the blood pressure. In addition, the reinfection at the aortic stump site promotes tissue fragility and may lead to rupture. Therefore, we believe that the long-term administration of antibiotic therapy and strict blood pressure control are necessary.

In the present case, severe infection around the stent-graft made it impossible to predict the safety of the intrapelvic organs during aortic clamping or the leg clamping tolerance time, and EAB was selected to prevent repeated infection of the prosthetic graft after replacement. It will be necessary to continue monitoring this patient carefully regarding long-term patency and complications. The patient’s progression has been favorable to date.

## Conclusion

Stent-graft removal and EAB were performed for stent-graft infection, and the outcome was favorable. An in situ technique or EAB is selected for vascular reconstruction after stent-graft removal to treat stent-graft infection. However, EAB may be one of the appropriate therapies in cases of severe intraperitoneal infection.
